# A tunable color filter using a hybrid metasurface composed of ZnO nanopillars and Ag nanoholes

**DOI:** 10.1039/d2na00286h

**Published:** 2022-07-26

**Authors:** Yicheng Wang, Weikai Huang, Yu-Sheng Lin, Bo-Ru Yang

**Affiliations:** School of Electronics and Information Technology, Sun Yat-Sen University Guangzhou 510006 China linyoush@mail.sysu.edu.cn yangboru@mail.sysu.edu.cn

## Abstract

We propose the design of symmetrical and asymmetrical tunable color filters (TCFs) by using hybrid metasurface nanostructures in the visible wavelength range. They are composed of circular zinc oxide (ZnO) nanopillars and silver (Ag) nanoholes on a silica substrate. These TCFs exhibit ultrahigh transmission intensity over 90%, different tuning ranges, and polarization-dependent/independent characteristics. By changing the distance between the ZnO nanopillars and silica substrate, the resonant wavelength of TCFs could be tuned remarkably. Moreover, we also demonstrate the stability of TCFs under different disturbances and angles of incident light. Furthermore, the resonant wavelengths are red-shifted by increasing the ambient refraction index. TCFs exhibit great tunability and ultrahigh transmission intensity up to 100%. This design opens up an avenue to widespread optoelectronic applications, such as ultrahigh resolution color displays, high-efficiency biosensors, pressure sensors, and selective color filters.

## Abstract 2

Hybrid metasurface nanostructures composed of ZnO nanopillars and Ag nanoholes exhibit color tunability.

## Graphical abstract

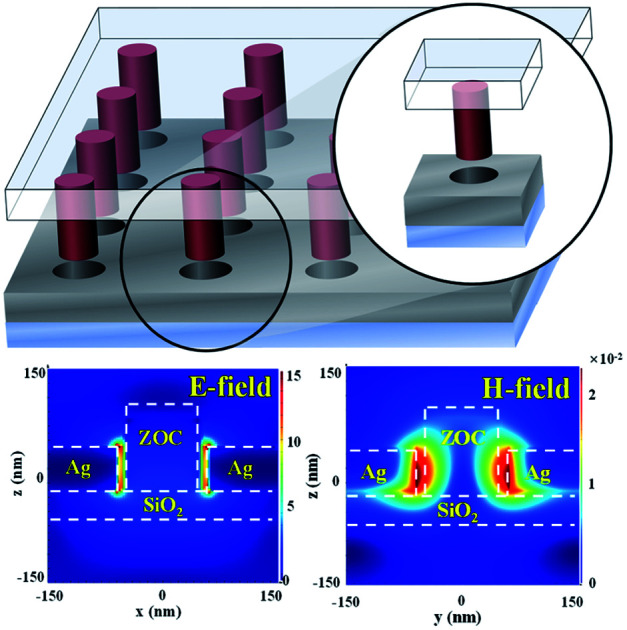

## 1. Introduction

Since Ebbesen and co-workers first observed the intriguing phenomenon of extraordinary optical transmission (EOT) from subwavelength apertures fabricated in metallic films in the late 1990s,^1,2^ it has generated considerable interest and has been studied intensively. Substantial designs have been presented to realize the manipulation of electromagnetic waves.^3–8^ The EOT effect is correlated to the resonant excitation of surface plasmon resonance (SPR) due to the coupling of incident electromagnetic waves at the metal–dielectric interface and localized surface plasmon resonance (LSPR) related to the feature size of the subwavelength structure,^9^ where the transmission intensity is higher than the prediction made by classic diffraction theory.^10^ The resonances are strongly sensitive to the feature sizes of nanoapertures, including the shape, period, thickness, and composition of the metal thin-film.^4,11,12^ For example, there are a large number of studies that explored the coupling effects of electromagnetic waves to triangular,^13,14^ circular,^4,6,11,15–17^ rectangular,^18,19^ elliptical,^20^ grating,^21–24^ S-shaped^25^ and other irregular^26–30^ configurations. Among them, metamaterials such as silver (Ag), aluminum (Al), and gold (Au) were widely used in the aforementioned investigations and showed great electromagnetic characteristics. Ag material is attractive for its low dielectric loss, relatively high plasma frequency, and high plasmonic quality factor, which can be obtained from1
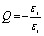
where *ε*_r_ and *ε*_i_ are the real and imaginary parts of the dielectric function of Ag material. Alternatively, Au material is popular for its outstanding plasmonic performance and chemical stability. Al material has also gained ample interest as a low-cost and sustainable material. Previous studies usually suffer from drawbacks such as low transmission intensity^16,22,25^ and poor tunability.^27,31–33^ For example, Balaur *et al.* proposed a tunable, polarization-controlled color palette produced from nanoscale plasmonic pixels; it is composed of cross-shaped nanoaperture arrays in a Ag thin film mounted on a silica substrate. Continuously tunable characteristics in the visible wavelength range is realized by simply adjusting the period of the cross-shaped apertures.^34^ However, the transmission intensity is unsatisfying (<30%), and it is hard to manipulate the resonances once the geometric parameters are fixed. In recent years, the micro–electro-mechanical system (MEMS) technique has become a preferable candidate method to obtain reconfigurable optoelectronic devices,^35–37^ enabling the manipulation of electromagnetic waves and providing possibilities for color filters with better turnability. It also possesses remarkable advancements, including a miniaturized size and fast response time.^38^ With the help of the MEMS technique, the nanostructures can be controlled to make the metamaterial device more flexible and applicable and possess outstanding tunability to enhance the transmission intensity. These advancements enable the metamaterial design to reach the requirement of applications, such as sensors, filters, ultrahigh-resolution, and anti-counterfeit displays.

In this study, we propose three designs of continuously tunable color filters (TCFs) using hybrid metasurface nanostructures in the visible wavelength range. They are composed of circular zinc oxide (ZnO) nanopillars and Ag nanoholes on a silica substrate. Recently, ZnO has attracted interest because of its stability, durability, electrochemical activity, high electron communication features and heat resistance. It has been used in biosensors,^39^ energy collection^40^ and other applications.^41^ For convenience, the configurations of ZnO nanopillars on the circular and elliptical Ag nanoholes are denoted as TCF-1 and TCF-2, respectively. TCFs exhibit ultrahigh transmission intensities (>90%) and great tunability by simply expanding the gap between the ZnO nanopillars and silica substrate. TCF-1 exhibits the polarization-insensitive characteristic with a tuning range of 75 nm from the wavelength of 488 nm to 563 nm. By breaking the symmetry of circular Ag nanoholes and changing the period of Ag nanoholes along the *y*-axis direction, *i.e.*, the TCF-3 configuration, it exhibits a greater tuning range spanning the entire visible spectrum in transverse electric (TE) and transverse magnetic (TM) modes. Moreover, the influences of polarization angles and surrounding environments on the transmission spectra are also discussed. The resonances of asymmetric designs can be tuned between two different resonant wavelengths by changing the polarization angle. Furthermore, the stability of TCFs is demonstrated by bending the ZnO nanopillars or shifting them along the *x*-axis direction. The resonances are maintained very well at different angles of incident electromagnetic waves. This work paves the way to widespread optoelectronic applications, such as sensors, optical filters, and ultrahigh-resolution and anti-counterfeit displays.^42–49^

## 2. Materials and methods

**Fig. 1:**
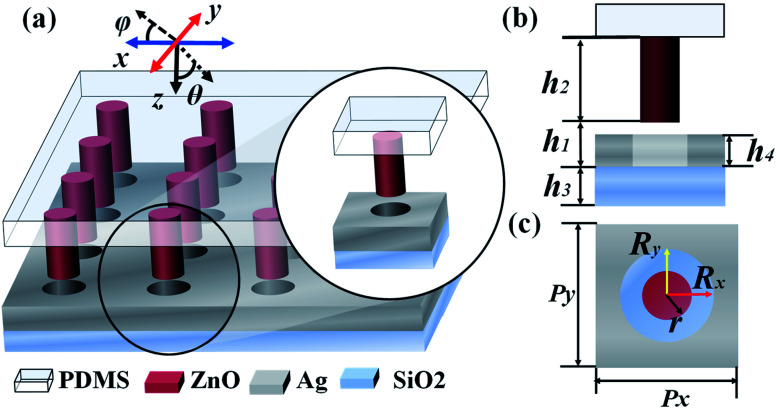
Schematic diagrams of the (a) 3D TCF-1 array, (b) cross-sectional view, and (c) top view of the TCF-1 unit cell, respectively.

Fig. 1(a)–(c) show the schematic diagrams of the 3D TCF-1 array, cross-sectional view, and top view of the TCF-1 unit cell, respectively. Ag thin-film with nanoholes is fabricated on a silica substrate, and then ZnO nanopillars are aligned and grown on the surface of Ag nanoholes. The PDMS layer is finally encapsulated on the top of the sample surface. The TE-polarized and TM-polarized waves are defined along the *x*- and *y*-axis directions, as highlighted by the blue and red arrows in Fig. 1(a), respectively. The polarization and incident angles are defined as *φ* and *θ*, respectively. The gap between the ZnO nanopillars and silica substrate, the thickness of the ZnO nanopillars, the thickness of the silica substrate, and the thickness of the Ag thin-film are defined as *h*_1_, *h*_2_, *h*_3_, and *h*_4_, respectively as shown in Fig. 1(b), where *h*_1_ is variable and *h*_2_ and *h*_3_ are kept as constants as *h*_2_ = 120 nm and *h*_3_ = 40 nm. The radius of ZnO nanopillars, the TCF period and the radius of Ag nanoholes along *x*- and *y*-axis directions are denoted as *r*, *P*_*x*_, *P*_*y*_, *R*_*x*_, and *R*_*y*_, respectively as shown in Fig. 1(c). The initial values are defined as *P*_*x*_ = *P*_*y*_ = 300 nm and *R*_*x*_ = *R*_*y*_ = 60 nm, *r* = 50 nm, respectively. The electromagnetic responses of TCFs are simulated using Lumerical Solution’s three-dimensional finite-difference time-domain (FDTD) based numerical simulations. The propagation direction of incident light is perpendicular to the *x*–*y* plane. Periodic boundary conditions are adopted in the *x*- and *y*-axis directions and a perfectly matched layer (PML) boundary condition is applied in the *z*-axis direction. The transmission of the incident wave is calculated by using a monitor set under the bottom side of the proposed TCFs.

## 3. Simulation results and discussions

**Fig. 2:**
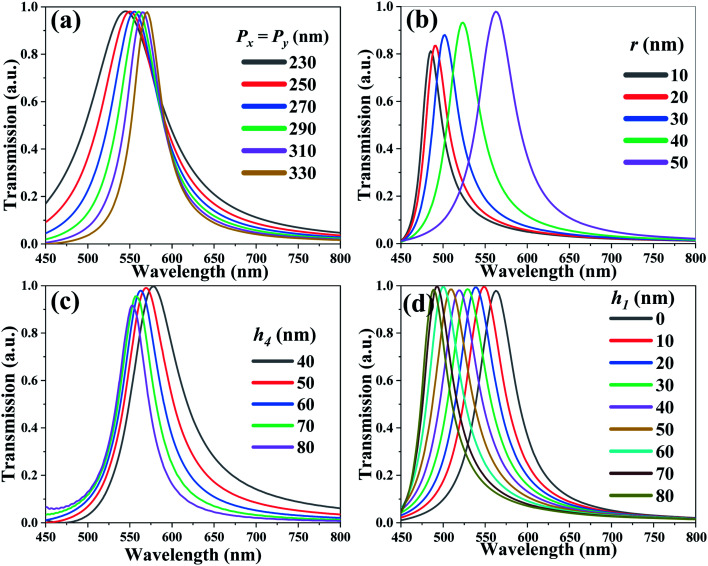
Transmission spectra of TCF-1 with different values of (a) periods (*P*_*x*_ = *P*_*y*_) from 230 nm to 330 nm, (b) *r* from 10 nm to 50 nm, (c) thickness of Ag nanoholes (*h*_3_) from 40 nm to 80 nm, and (d) *h*_1_ from 0 nm to 80 nm under normal TE-polarized incident light.

Fig. 2(a) shows the transmission spectra of TCF-1 with different period values. Herein, the values of *P*_*x*_ and *P*_*y*_ are changed simultaneously from 230 nm to 330 nm under normal incident TE-polarized light, while keeping *h*_1_ = 0 nm and other parameters as constants. There is a single resonance around the wavelength of 550 nm. The resonances are red-shifted by 25 nm from the wavelength of 545 nm to 570 nm by increasing the TCF period from 230 nm to 330 nm. The full width at half maximum (FWHM) of resonances is decreased from 103 nm to 41 nm, which is accompanied by an increment in the quality (Q) factors of the resonances from 5.29 to 13.90. The TCF periods are kept as *P*_*x*_ = *P*_*y*_ = 300 nm for further investigations considering the *Q*-factor and tuning range of resonance in the following discussions. Fig. 2(b) shows the transmission spectra of TCF-1 with different *r* values from 10 nm to 50 nm by keeping *h*_1_ = 0 nm and other parameters as constants. By increasing the *r* value, the resonances are red-shifted by 77 nm from the wavelength of 485 nm to 563 nm and the transmission intensities are increased from 81% to 97%. This is attributed to the intensification of SPR, which is related to the coupling field at the metal-dielectric interface. Thus, the *r* value is defined as 50 nm for further investigations considering the transmission intensity in the following discussions. This ultrahigh transmission intensity is attributed to the SPR due to the coupling effect of the incident electromagnetic wave at the metal–dielectric interface and LSPR related to the circular geometry of the Ag nanohole array.^9^Fig. 2(c) shows the transmission spectra of TCF-1 with different *h*_4_ values while keeping *h*_1_ = 0 nm and other parameters as constants. By increasing the *h*_4_ value from 40 nm to 80 nm, the resonances are blue-shifted by 26 nm from the wavelength of 578 nm to 552 nm, and the transmission intensities are decreased slightly from 100% to 92%. The FWHM values are changed from 69 nm to 44 nm accompanied by the increment of *Q*-factors from 8.36 to 12.55. Considering the tradeoff of the transmission intensity and *Q*-factor, the *h*_4_ value is defined as 60 nm for further investigations. Fig. 2(d) shows the transmission spectra of TCF-1 with different *h*_1_ values from 0 nm to 80 nm while keeping other parameters as constants. The resonances are blue-shifted by 75 nm from the wavelength of 563 nm to 488 nm. The transmission intensities are quite stable at an average of 99%, and the averaged FWHM values and *Q*-factors are 50 nm and 10.61, respectively. These results indicate that the *h*_1_ parameter plays a critical role in determining the optical transmission properties of TCF-1. This is because when the ZnO nanopillars are elevated (*h*_1_ > 0), the Fabry–Perot (F–P) cavity is enlarged between the ZnO nanopillars and the silica substrate surface, which is an optical resonator typically made from parallel facing mirrors in which a light field can be selectively enhanced through resonance. In this case, the patterned Ag thin-film and ZnO nanopillars can be considered as a F–P resonator. This F–P cavity is formed to generate F–P resonance, which corresponds to the reflection resonance of TCF and can be determined from^16,50^2
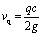
where *q* is the mode index, *g* is the length of the F–P cavity, and *c* = *c*_0_/*n* where *c*_0_ is the speed of light in vacuum and *n* is the refraction index of the medium. According to the simulation results, the design of TCF does not absorb electromagnetic energy. Thus, the transmission can be approximately expressed as *T* = 1 − *R*. It means that the red-shift of reflection resonance will result in the blue-shift of transmission resonance. Therefore, the coupling effects of SPR, LSPR, and F–P resonances result in blue-shift characteristics and ultra-high transmission intensities. This is the reason for the influence of the *h*_1_ parameter on the resonance. According to the above-mentioned results, the resonances of TCF-1 can be manipulated actively by simply changing the gap between ZnO nanopillars and Ag nanoholes, which shows good potential in pressure sensors, high-resolution displays, and tunable color filters.

**Fig. 3:**
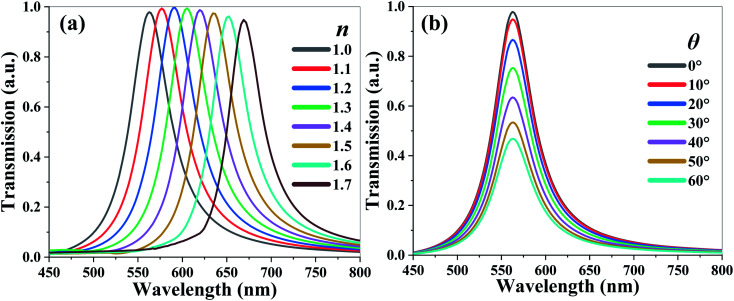
Transmission spectra of TCF-1 (a) exposed to the ambient environment with different *n* values from 1.0 to 1.7 under normal TE-polarized incident light and (b) with different *θ* values from 0° to 60° in TE mode.

Fig. 3(a) shows the transmission spectra of TCF-1 exposed to the ambient environment with different refraction index (*n*) values from 1.0 to 1.7 while keeping *h*_1_ = 0 nm and other parameters as constants under normal TE-polarized incident light. By increasing the *n* value, the resonances are red-shifted by 106 nm from the wavelength of 562 nm to 668 nm with an averaged transmission intensity of 98%. It is because nanohole arrays confine most of the near field energies. The strong transmission resonances are highly dependent on the change of the environmental *n* value. The averaged FWHM values and Q-factors are 50 nm and 12.05, respectively. The sensitivity (*S*) of resonance to the *n* value can be expressed as3
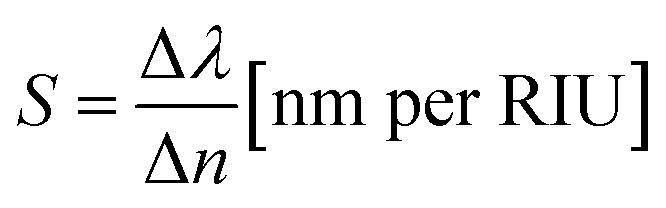
where Δ*λ* is the resonance shift and Δ*n* is the variation of the surrounding environment. The sensitivity is calculated to be 151.4 nm per RIU. The figure of merit (FOM) is defined as *S* divided by the FWHM, which is a widely accepted metric for characterizing the performance of a refraction index sensor. The FOM value of TCF-1 is calculated to be 3.05. The performance of TCF-1 exposed to different surrounding environments proves that the proposed TCF-1 can be used as a high-efficient refraction index sensor. One of the strict requirements for the application of color filters in display technology is that the color filters must be angle insensitive.^17^ To prove this characteristic of TCF-1, the transmission spectra of TCF-1 at different angles of incident TE-polarized light (*θ*) are depicted in Fig. 3(b). The resonant wavelengths remained unchanged except for the resonant intensities because the effect of LSPRs is independent of the incident angle.^48^ The transmission intensities under the conditions of *θ* = 0°, *θ* = 30° and *θ* = 60° are 98%, 75%, and 47%, respectively. The variation of transmission intensity is within 50% by changing the angle of incident light to 60°.

**Fig. 4:**
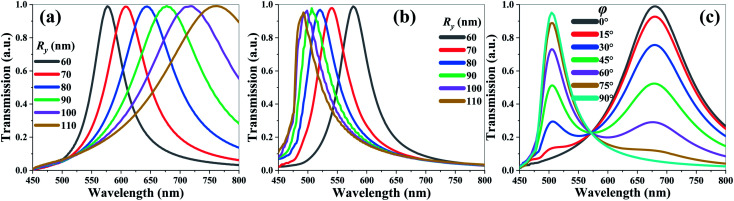
Transmission spectra of TCF-2 with different *R*_*y*_ values in (a) TE and (b) TM modes at normal incidence. (c) Transmission spectra of TCF-2 with different *φ* values from 0° to 90° under the condition of *R*_*x*_ = 60 nm and *R*_*y*_ = 90 nm at normal incidence.

This is because the tuning performance of TCF-1 is limited in the blue spectrum range (Fig. 2(d)), TCF-2 is proposed by enlarging the *R*_*y*_ value from 60 nm to 110 nm and keeping *R*_*x*_ as constant as 60 nm to broaden the tuning range of the resonant wavelength. Fig. 4(a) and (b) show the transmission spectra of TCF-2 with different *R*_*y*_ values under normal TE-polarized and TM-polarized incident lights, respectively. As shown in Fig. 4(a), the resonances of TCF-2 are significantly red-shifted by 182 nm from the wavelength of 579 nm to 761 nm in TE mode. The FWHM values under the conditions of *R*_*y*_ = 60 nm and *R*_*y*_ = 90 nm are 61 nm and 138 nm, respectively. As shown in Fig. 4(b), the resonances of TCF-2 are blue-shifted by 91 nm from the wavelength of 579 nm to 488 nm in TM mode. The FWHM values under the conditions of *R*_*y*_ = 60 nm and *R*_*y*_ = 90 nm are 59 nm and 56 nm, respectively. These results indicate that TCF-2 exhibits different shifting trends in TE and TM modes by increasing *R*_*y*_. It can be observed that the variation of FWHM values is stable in TM mode compared to that in TE mode. Herein, the *R*_*y*_ value is defined as 90 nm for next discussions of enhancing the tuning range of resonance. It is expected that TCF-2 possesses the polarization-sensitive characteristic, because LSPR is strongly related to the feature size of the subwavelength structure. Increasing the *R*_*y*_ value can affect the excitation of LSPR. Meanwhile, the polarization state of incident light can also significantly affect the SPR condition, especially in asymmetrical nanoapertures.^14^ The influence can be understood through the propagation direction of the surface plasmons (SP) mode, which is parallel to the electric field.^20^ Considering that the coupling effect in and out of the SP modes occurs at the edges of the nanoholes, the resonance generated from the periodic nanohole array is aligned with the optical polarization. The variation of the *R*_*y*_ parameter causes the changes of LSPR and SP, and then results in the resonance shift. The transmission spectra of TCF-2 with different polarization angles (*φ*) from 0° (*i.e.*, TE mode) to 90° (*i.e.*, TM mode) under the conditions of *R*_*x*_ = 60 nm and *R*_*y*_ = 90 nm under normal incident light are shown in Fig. 4(c). The resonance at the wavelength of 680 nm can be attenuated by increasing the *φ* value. The transmission intensity is modified from 99% to 5% by changing the *φ* value from 0° to 90°. Meanwhile, the resonance at the wavelength of 504 nm is enhanced from 7% to 96%. These results indicate that TCF-2 possesses an ultrahigh color modulation depth, which can be switched between green and red colors by changing the incident polarization angle. It implies that the design of TCF-2 provides potential as a high-efficient color switch.

**Fig. 5:**
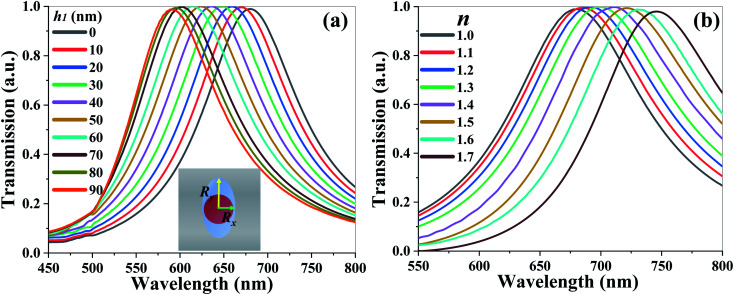
Transmission spectra of TCF-2 (a) with different *h*_1_ values from 0 nm to 90 nm and (b) exposed to the ambient environment with different *n* values from 1.0 to 1.7 under the condition of *R*_*x*_ = 60 nm, *R*_*y*_ = 90 nm under normal TE-polarized incident light.

Fig. 5(a) shows the transmission spectra of TCF-2 with different *h*_1_ values from 0 nm to 90 nm under the conditions of *R*_*x*_ = 60 nm and *R*_*y*_ = 90 nm while keeping other parameters as constants under normal TE-polarized incident light. The resonances are blue-shifted by 91 nm from the wavelength of 679 nm to 590 nm by increasing the *h*_1_ value. The FWHM values are quite stable and the averaged FWHM values and *Q*-factors are 132 nm and 4.77, respectively. TCF-2 can be tuned to undergo color switching between yellow and red spectra by changing the *h*_1_ value. Fig. 5(b) shows the transmission spectra of TCF-2 exposed to the ambient environment with different *n* values from 1.0 to 1.7 under the condition of *R*_*x*_ = 60 nm and *R*_*y*_ = 90 nm under normal TE-polarized incident light. The resonances are red-shifted by 68 nm from the wavelength of 679 nm to 747 nm by increasing the *n* value. The sensitivity of resonance to the *n* value is calculated to be 97.14 nm per RIU and the corresponding FOM is 0.74.

**Fig. 6:**
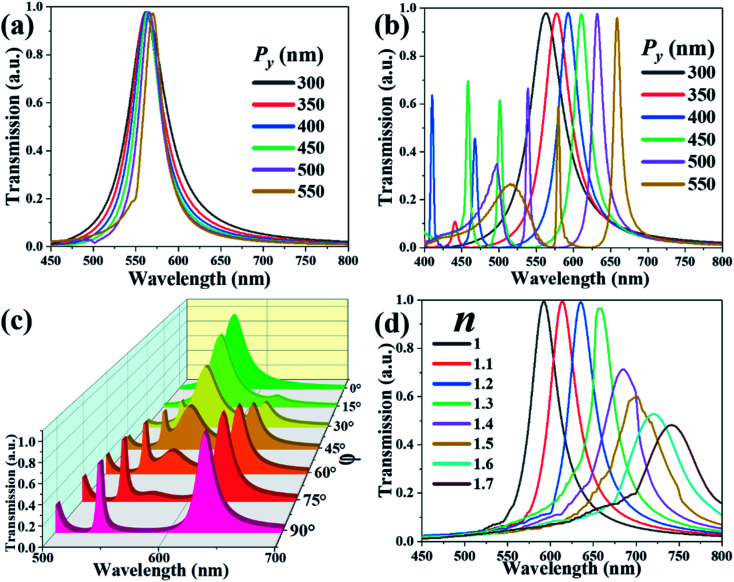
Transmission spectra of TCF-2 with different *P*_*y*_ values from 300 nm to 550 nm at normal incidence in (a) TE and (b) TM modes. Transmission spectra of TCF-2 (c) with different *φ* values at the normal incidence and (d) exposed to the ambient environment with different *n* values under the condition of *P*_*x*_ = 300 nm and *P*_*y*_ = 500 nm under normal TE-polarized incident light.

To endow the design with greater versatility and tunability, TCF-3 is proposed by extending the TCF period along the *y*-axis direction. TCF-3 is polarization-sensitive due to its asymmetric configuration. Fig. 6(a) and (b) show the transmission spectra of TCF-3 with different *P*_*y*_ values from 300 nm to 550 nm while keeping *P*_*x*_ as constant at 300 nm at the normal TE- and TM-polarized incident lights, respectively. As shown in Fig. 6(a), the resonances of TCF-3 shifted slightly in TE mode. The variation is less than 10 nm. In addition, the FWHM values become narrower from 54 nm to 29 nm, and the *Q*-factors increase from 10.37 to 19.58. Fig. 6(b) shows that the resonances are blue-shifted by 95 nm from the wavelength of 562 nm to 657 nm. The FWHM values are changed from 52 nm to 10 nm. TCF will generate second and third resonances when the *P*_*y*_ is larger than 400 nm. These subordinate resonances are also red-shifted by increasing the *P*_*y*_ value. These results indicate that the resonances of TCF-3 are affected by the periodic change along the polarized direction of incident light. Transmission spectra of TCF-3 with different polarization angles (*φ*) from 0° to 90° under the condition of *P*_*x*_ = 300 nm and *P*_*y*_ = 500 nm are depicted in Fig. 6(c). When *φ* equals 0°, there is a single-resonance at the wavelength of 562 nm. By increasing the *φ* value from 0° to 90°, the resonance becomes weaker and vanishes, and the resonances at wavelengths of 631 nm and 539 nm become more intensive, indicating that TCF-3 can operate as a light switch between red and green light simply by varying its polarization angle. Fig. 6(d) illustrates the transmission spectra of TCF-3 with different values of the environmental refraction index (*n*) under the condition of *P*_*x*_ = 300 nm and *P*_*y*_ = 500 nm under normal incident TE-polarized light. The resonances are red-shifted by 179 nm from the wavelength of 562 nm to 741 nm by increasing the *n* value. The transmission intensities are remarkably reduced when *n* is greater than 1.4. The sensitivity is 255.7 nm per RIU, which outperforms the two former designs. The optimized TCF-3 can be further used as an outstanding environmental sensor.

**Fig. 7:**
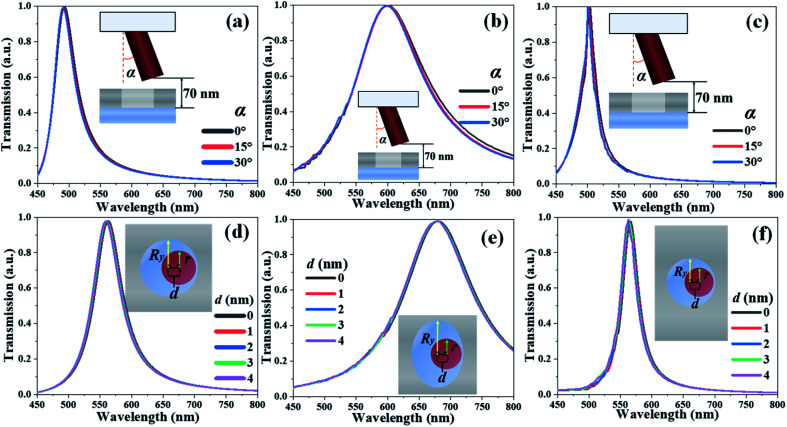
Transmission spectra of (a) and (d) TCF-1, (b) and (e) TCF-2 and (c) and (f) TCF-3 with different (a–c) *α* values and (d–f) *d* values while keeping other parameters as constants under normal TE-polarized incident light.

In order to demonstrate the stability of TCF-1, TCF-2, and TCF-3, the rotation angle and displacement of ZnO nanopillars along the *x*-axis direction are defined as *α* and *d*, respectively, to simulate the variations and disturbances during the real operation as illustrated in Fig. 7. Fig. 7(a)–(c) show the transmission spectra of TCF-1, TCF-2, and TCF-3 with different *α* values from 0° to 30° while keeping *h*_1_ = 70 nm under normal incident TE-polarized light, respectively. Fig. 7(d)–(f) show the transmission spectra of TCF-1, TCF-2, and TCF-3 with different *d* values from 0 nm to 4 nm while keeping *h*_1_ = 0 nm under normal incident TE-polarized light, respectively. The main resonances are SPR and LSPR, and F–P resonance is adopted to enable the tunability of TCFs. By changing *α* and *d* values, that does not affect the behaviors of SPR and LSPR, the geometry of F–P cavity is also fundamentally unchanged. These results indicate that the TCFs are stable by changing *α* and *d* parameters by pressing, bending, and shocking the TCF devices. TCF devices exhibit extraordinary stability under interferences and also allow the tolerance to manufacturing imperfection.

**Fig. 8:**
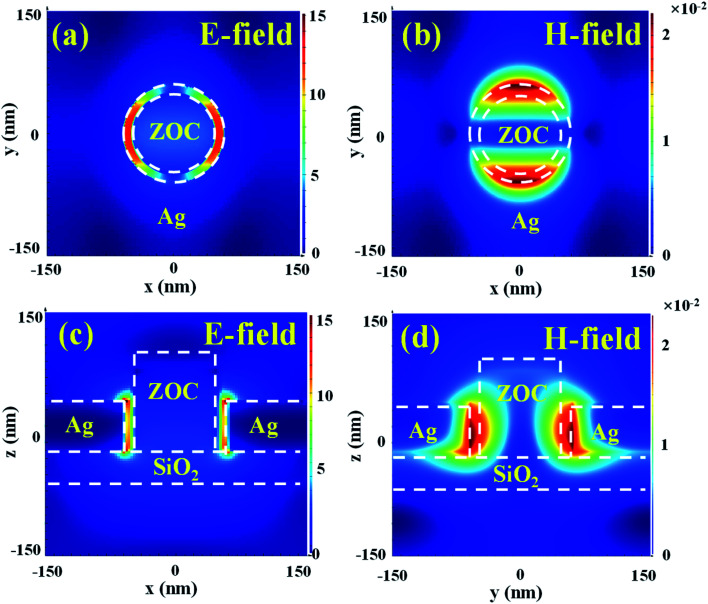
(a) and (b) Top views and (c) and (d) cross-sectional views of (a) and (c) *E*- and (b) and (d) *H*-field distributions of TCF-1 in TE mode.

These ultrahigh transmission intensities of TCFs are attributed to the theories of SPR and LSPR due to the coupling effects of incident electromagnetic waves at the metal–dielectric interface and the geometrical parameters of circular Ag nanoholes.^9,49^ The conduction electron charge density and the corresponding electromagnetic field can undergo plasmon oscillations, which will propagate along the metal surface and are known as surface plasmon polaritons (SPPs),^51^ which can overcome the conventional diffraction limit and manipulate the propagation direction of incident light on the subwavelength scale. For periodic nanostructures in a metal thin-film, the dispersion equation of resonance for different Bloch modes of SPPs at normal incidence is widely used,^2,49^ which is expressed as4
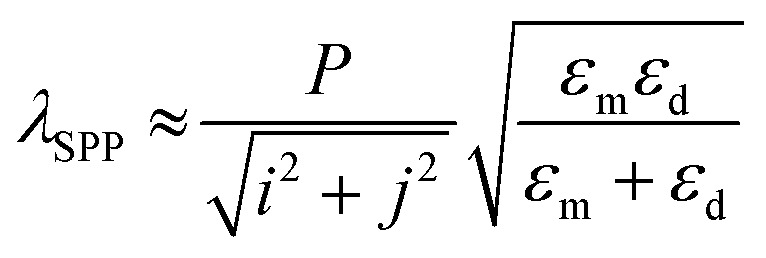
where *P* is the array periodicity, *i* and *j* are integers corresponding to the scattering orders from the aperture array, and *ε*_m_ and *ε*_d_ are the permittivity of the metal and dielectric medium, respectively. It can be used to approximately estimate the resonance in this study. When *ε*_d_ is increased, the value of the square root in the equation increases and then the value of *λ*_spp_ increases. The red-shifting characteristics of TCFs can be explained by increasing the *n* value. To further understand the formation of the resonance and investigate the function of ZnO nanopillars, we take TCF-1 as an example to analyze its electromagnetic field distributions. The geometrical parameters of TCF-1 are *P*_*x*_ = *P*_*y*_ = 300 nm, *R*_*x*_ = *R*_*y*_ = 60 nm, *r* = 50 nm, *h*_1_ = 0 nm, and *h*_4_ = 60 nm. The absolute electric (*E*) and magnetic (*H*) field distributions of TCF-1 at the TE resonance are depicted in Fig. 8. Fig. 8(a) and (b) illustrate the top views of *E*- and *H*-field distributions of TCF-1 at the wavelength of 563 nm, respectively. In Fig. 8(a), the inner and outer white dotted lines are the outlines of the ZnO nanopillar and Ag nanohole, respectively. The *E*-field energy is fundamentally confined between the gap of the ZnO nanopillar and Ag nanohole along the *x*-axis direction. It implies the excitation of LSPR, which shows dipolar characteristics. The white dotted lines depicted in Fig. 8(b) are the outlines of the ZnO nanopillar and Ag nanohole, respectively. The *H*-field energy is mainly confined between the gap of the ZnO nanopillar and Ag nanohole along the *y*-axis direction, which agrees well with the result shown in Fig. 8(a). Fig. 8(c) and (d) show the cross-sectional views of absolute *E*- and *H*-field distributions of TCF-1 at the TE resonance of 563 nm. As shown in Fig. 8(c), the *E*-field energy is mainly accumulated in the Ag nanohole, which indicates the excitation of SPR between the metal and dielectric layers. It can be concluded that ZnO nanopillars can confine the *E*-field energy between the gap of the ZnO nanopillar and Ag nanohole. Compared with the result in Fig. 8(b), the *H*-field energy is mainly accumulated within the gap of the ZnO nanopillar and Ag nanohole along the *y*-axis direction as shown in Fig. 8(d). The theoretical model in eqn (3) only accounts for the propagating surface plasmons, while the localized surface plasmons can also be excited during the process.^52^ The *E*- and *H*-field distribution indicate that the LSPR excites a dipolar resonance with a strong near-field enhancement. It demonstrates that the resonant wavelength is strongly dependent on the change in the surrounding refraction index, which can explain the frequency shift caused by the *n* parameter. Therefore, the existence of ZnO nanopillars results in stronger resonances and the enhancement of transmission intensity, and the ultrahigh transmission intensities can be attributed to the coupling effects of SPR and LSPR.

## 4. Conclusions

In conclusion, we propose three designs of TCFs by using hybrid metasurface nanostructures composed of ZnO nanopillars and Ag nanoholes to investigate the superior electromagnetic properties in the visible frequency range. TCF-1 and TCF-2 exhibit great tunability by simply changing the gap between the ZnO nanopillars and silica substrate. TCF-1 possesses the characteristics of polarization independence, angle insensitivity, and ultrahigh transmission intensity. By changing the TCF period and tailoring the circular shape into an elliptical shape, TCF-2 and TCF-3 show different tuning ranges and polarization-dependent characteristics. Moreover, TCF-3 exhibits color switching characteristics. The designs of TCFs can be designed properly to possess great tunability in the entire visible wavelength range by tailoring the geometrical parameters. TCFs exhibit remarkable stability and provide good durability and tolerance to the interference of the external environment. When TCFs are exposed to the different ambient environments, their resonances can be red-shifted by increasing the environmental refraction index. The sensitivities of TCF-1, TCF-2, and TCF-3 are 151.4 nm per RIU, 97.14 nm per RIU, and 255.7 nm per RIU, respectively. These TCFs are desirable for widespread optoelectronic applications, such as high-efficiency biosensors, pressure sensors, selective color filters, high-resolution displays, refractive index sensors, *etc.*

## Data availability

The data that support the findings of this study are available from the corresponding author upon reasonable request.

## Conflicts of interest

The authors declare no conflicts of interest.

## Supplementary Material
